# Quality of life assessment in breast cancer patients during palliative treatment in Indonesia

**DOI:** 10.1093/eurpub/ckac131.205

**Published:** 2022-10-25

**Authors:** D Gayatri, L Efremov, A Wienke, R Mikolajczyk, EJ Kantelhardt

**Affiliations:** 1 Institute for Medical Epidemiology, Martin-Luther-University Halle-Wittenberg, Halle, Germany; 2 Department of Epidemiology, Faculty of Public Health, Universitas Indonesia, Depok, Indonesia; 3 Department of Radiation Oncology, Martin-Luther-University Halle-Wittenberg, Halle, Germany; 4 Department of Gynecology, Martin-Luther-University Halle-Wittenberg, Halle, Germany

## Abstract

**Background:**

This study aimed to prospectively assess quality of life (QOL), QOL domains, and pain severity in advanced stage breast cancer patients during palliative oncology treatment in Indonesia.

**Methods:**

Advanced stage breast cancer patients > 18 years (n = 160) who began palliative oncology treatment were enrolled in the study using convenience sampling. They completed the EORTC QLQ-C15-PAL questionnaire and pain severity (Visual Analogue Scale, VAS) score at three-time points (baseline (T0), three-(T1) and six-months (T2) follow-up). The repeated measures analysis of variance (ANOVA) model was used to assess the QOL, QOL domains, and pain severity changes over time adjusted for age, place of residence, marital status, and Karnofsky Performance Status score at baseline. We classified the change over time in three qualitative groups (deterioration, improvement, or trivial/no difference). We considered it clinically relevant if patients had a 10-point difference.

**Results:**

The mean age of included patients (n = 159) was 50 years. Most lived in an urban area (72.3%), had low education (71.7%), and were married (81.8%). The repeated measures ANOVA showed that the QOL score, emotional functioning, fatigue, dyspnea, appetite loss, constipation, and VAS pain score remained stable over the 6-months period. In contrast: physical functioning declined (medium to large deterioration (-19.8 (95% CI -27.2 to -12.5)) between T0 to T2), however there was an improvement in the insomnia domain (medium improvement (-13.4 (95% CI -19.9 to -6.9)) between T0 to T2).

**Conclusions:**

Our findings indicated that advanced stage breast cancer patients adapted well to palliative oncology treatment over six months of observation. There was deterioration in physical functioning, but improvement in insomnia. However, more attention is needed from clinicians to achieve improvement in the overall QOL score and specific QOL domains.

**Key messages:**

• Focusing on improvement overall QOL score and specific QOL domains will lead to better advanced stage breast cancer patients’ satisfaction and care.

• Information is limited on palliative treatment satisfaction in low and lower middle-income countries, therefore this study has important impact on further policy considerations in Indonesia.

